# SES Differences in Children’s Argumentative Production

**DOI:** 10.5964/ejop.v16i2.1665

**Published:** 2020-05-29

**Authors:** Maia J. Migdalek, Celia R. Rosemberg

**Affiliations:** a National Council of Scientific and Technical Research, Buenos Aires, Argentina; b University of Buenos Aires, Buenos Aires, Argentina; Department of Psychology and Counselling, Webster University Geneva, Geneva, Switzerland

**Keywords:** children, disputes, argumentation, connector, SES differences

## Abstract

Recent studies have examined the argumentative strategies used by young children in everyday situations as well as in experimental settings. However, differences in argumentative production as a function of Socio-Economic Status (SES) have been minimally explored. This study aims to analyze eventual differences regarding social group in the use of argumentative strategies and connectors marking causal and adversative relationships within these strategies. The corpus is 615 disputes occurred during play situations in the homes of 39 4-year old children living in Buenos Aires, Argentina: 453 of mid SES children and 162 of low SES. Argumentative strategies were codified using a system of inductively derived categories: a) the reiteration of the child’s point of view; b) the narration of previous experiences; c) the anticipation of courses of action; d) generalization; e) the description of the characteristics of an object, event or internal state; f) referencing authority; g) the mitigation of the point of view; h) providing an alternative proposal. Results show that in both social groups the use of an argumentative strategy to sustain the point of view predominates over merely stating the point of view. Additionally, we found significant differences in a) Reiteration strategy, with the low SES group showing a greater use of this strategy and b) Generalization and Description strategies, with the mid SES children employing these ones more frequently. Regarding the connectors, significant differences were only detected in the use of consecutive and adversative markers. The mid SES group showed a greater use of these particular connectors.

Recently, various studies have shown that young children, starting at age three, are capable of defending their points of view through some kind of argumentative formulation (Bernard, Mercier, & Clément, 2012; Crespo, 1995; Dunn & Munn, 1987; Eisenberg, 1987; Köymen, Rosenbaum, & Tomasello, 2014; Migdalek & Rosemberg, 2013; Migdalek, Rosemberg, & Santibáñez Yáñez, 2014; Migdalek, Santibáñez Yáñez, & Rosemberg, 2014; Pontecorvo & Arcidiacono, 2010; Wyman, Rakoczy, & Tomasello, 2009). These studies addressed child argumentation by using several methodologies and theoretical frameworks (Cobb-Moore, Danby, & Farrell, 2008, 2009; Crespo, 1995; Eisenberg, 1987; Kline, 1998; Kuhn, 1992; Peronard, 1991; Pontecorvo & Arcidiacono, 2010; Stein & Albro, 2001; Voss & Van Dyke, 2001; Zadunaisky Ehrlich & Blum-Kulka, 2010). Whilst in some studies children’s argumentation is elicited through experimental or quasi experimental situations, others study child argumentation in naturalistic settings. Among these, Eisenberg’s (1987) classic paper, conceptualizes the argumentative strategies employed by young girls in disputes with their parents and peers. She identifies the following strategies: insistence, verbal support, mitigation, appealing to another individual, threatening, use of verbal abuse, and offers to compromise. Peronard’s (1991) four-year longitudinal study with five Chilean children analyzes children’s utterances used to persuade their adult interlocutor, generally the mother. These kinds of utterances might constitute the precursor of argumentative discourse. These utterances initiate in the dialogical sequence, triggered by adults and later, in development, take form in an autonomous way. They mostly consist of argumentative points regarding actions, related to the material world, the cultural world, or the psychological world.

Dunn and Munn (1987), on their part, explored young children’s justifications in disputes, considering the differences with regard to the interlocutor, mother and older sibling. They carried out a longitudinal study with 43 children aged 18, 24, and 36 months, within the home context. The analysis reveals that at 36 months justifications were equally frequent with mother and sibling. In these justifications children appeal mainly to their own feelings, but they also draw on referencing social norms, particularly in disputes with their siblings. The other participants in the dispute, both the mother and the sibling, offered justifications, although the mothers did so more frequently. As children grow older, both mothers and siblings are more likely to anticipate eventual consequences of the actions as justifications for their points of view. Mothers and siblings draw on referencing social norms with similar frequency to each other. Thus, the authors maintain that not only the parents but also the siblings are potentially important agents of socialization for children during early childhood.

In another study, Goetz (2010) focuses on verbal justifications present in spontaneous conversations between children and adults. The author longitudinally follows up on four children (from 2:6 and 4:11) extracted from the CHILDES database. The analysis considers who produces the justification—parent or child—, the discursive context in which it appears and the age of the child. Results show an increasing tendency in the percentage of justification production on the part of the children, as a function of age. Nevertheless, this tendency is not observed in parent’s justifications. In relation to discursive contexts, conflicts appear to be important for the production of justifications between the ages of 2:6 and 2:11. By contrast, during the third year, questions stood out as promoters of justifications and between the ages of 4:0 and 4:11, the justifications are mostly produced as self-expansions.

Other studies addressed child argumentation preschool context. Zadunaisky Ehrlich and Blum-Kulka (2010) analyzed argumentative events in preschool children’s natural interactions. Results showed that children, in order to negotiate their social relationships, manage the argumentative event effectively employing rationally based strategies characteristic of adults. Nevertheless, they did not conceptualize the variety of strategies which configure child argumentation.

For their part, Cobb-Moore, Danby, and Farrell (2008, 2009) studied children’s justifications used to maintain a stance during play situations and other activities at preschool. In their first study (2008) they created a system that categorizes the content of the children’s justifications. The system is based on the rule of transferred ownership, the rule of first possession, rules associated with custodianship, and the rule of third-party verification. The authors point out that the justification of point of view in the dispute constitutes a practice which allows children to construct and/or reinforce their status within the group while, at the same time, it contributes towards maintaining social order in the classroom.

In their second study (2009) they analyze how young children manage social interaction in play situations. The authors identified four interactional practices: a) they claim possession of objects and play spaces; b) they appeal to pre-existing rules and to the social order to control interactions with their peers; c) they use language strategically to regulate the actions of those around them; and d) they create and employ membership categories to include or exclude others and also to control and participate in the ongoing interaction.

For their part, Pontecorvo and Arcidiacono (2010) found differences in the argumentative strategies employed by children according to context. They analyzed a narrative situation in the preschool context as well as mealtime in family settings. The qualitative analysis evidenced differences in the strategies employed by children in each context. The narrative at preschool stemmed from a conflict presented in a story. The children were asked to imagine different possible outcomes of this conflict. The analysis took children disputes regarding the resolution of the narrative conflict into account. In these disputes, the children defended their points of view via reasoning that displayed the possible negative consequences of the various hypothetical conditions and via the use of counterfactual forms. The authors highlight the role of the teacher in scaffolding this argumentative interaction through repetitions and reformulations of the children’s interventions. For their part, child-parent conversations during mealtimes at home children were characterized by parents’ explanations and justifications of everyday life rules through conditional structures and negative forms, similar to the counterfactual forms produced by the children at the preschool. In these mealtimes children also tend to use such structures. Thus, the authors maintain that these exchanges of practical reasoning imply an opportunity for children to develop and practice certain discursive resources.

Recently, Köymen, Rosenbaum, and Tomasello (2014) investigated 3 and 5-year-olds use of culturally common knowledge as a guarantee in order to justify their points of view within episodes of shared reasoning. They evaluated the children’s argumentative production whilst completing a task which entailed locating conventional and non conventional items in the context of a zoo. The results showed that when the episode included reasoning about a non conventional item, the children in both age groups explained the guarantee; but when the item was conventional, they would rely on the implicit guarantee. Also, a reasoning episode which included a large number of justifications was usually resolved in a consensual rather than a forced way. Results showed that 5-year-old children make the guarantees explicit more often than the 3 year olds, and that they tend to resolve reasoning episodes in a consensual way. An increment in social reasoning was evident between the ages of 3 and 5 as a consequence of social interaction and of their perception of the way in which their arguments are received by their interlocutors.

Kuhn (1992) studied the differences in primary school children’s argumentative performance considering their grade level and school experiences. Results showed differences in the argumentative performance of children from third, sixth, and ninth grades as well as differences related to the academic level of the school they attended. These findings coincide with what has been pointed out by other authors (Faigenbaum, 2012; Kline, 1998; Silvestri, 2001) who have indicated that the environment should promote argumentative competence in order for the children to develop it.

Another investigation carried out by Kline (1998) with mid and high Socio-Economic Status (SES) children in the second, fourth and sixth grades of primary school confirms this statement. Through a design in which she combines interviewing children with argumentative production tasks, Kline links the quality of children’s argumentation to previous experiences of children in persuasive interactions with others. The study showed a positive correlation between children’s argumentative skills and the exchanges in which the children had cooperated and argued previously. Kline concludes that when children have the opportunity to initiate and ponder arguments pose by others, and to participate equally in the resolution of disputes, they develop competence in persuasive argumentation.

In Spanish, the oppositional relationship in a dispute is indicated linguistically through the form “pero” (but). On the other hand, the consequence and cause relationship between the position and the argument may be marked linguistically through the connector “porque*”* (because). If, conversely, what is highlighted is a cause and consequence relationship the connector “por eso*”* (therefore) is employed. Various investigations from a developmental perspective identified an early use of these connectors by the end of a child’s second year (Braunwald, 1985; French & Nelson, 1985; Levy & Nelson, 1994; McCabe & Peterson, 1985, 1997). This does not imply, as Tomasello (2003) and Nelson (1996) maintain, that the appropriate use of the form in a particular context entails children’s complete understanding of its meaning. Initially, children may use connectors in discursive contexts in which they heard them previously; their pragmatic use in the situation may reflect only a partial dimension of the meaning. With regard to this, French (1989) sustains that, most likely, representations of general events or scripts construct a *cognitive context* in the framework of which the child is able to understand causal, conditional, and disjunctive relationships. It is within the framework of these cognitive contexts that children initially produce the connectors that explicitly textualize these relationships. A minimal understanding of the form is necessary for its use, but a semantic reorganization leading to complete comprehension only takes place through its use in interactive situations. A series of studies have described the use of connectors in dispute situations (Migdalek, Rosemberg, & Santibáñez Yáñez, 2014; Orsolini, 1993; Pellegrini, 1985; among others). Other studies have found that the justifications produced in validation contexts in which the point of view of another interlocutor is reinforced lead to a greater use of causal connectors than the ones produced in dispute contexts (Kyratzis, Ross, & Köymen, 2010).

This thorough review of the literature shows that previous research has mainly analyzed argumentative production elicited in experimental or quasi-experimental tasks. Those studies that have analyzed natural situations of argumentation have not controlled the type of activity in order to consider differences in SES, age and home or school environment where the activity takes place. Conversely, our studies about child argumentation focus on the analysis of 3 to 5-year-olds’ argumentative strategies in play situations at home or school. The results showed an early use of argumentative strategies, both verbal and non-verbal in order to regulate the joint action (Migdalek & Rosemberg, 2013, 2014; Migdalek, Santibáñez Yáñez, & Rosemberg, 2014; Migdalek, Rosemberg, & Santibáñez Yáñez, 2014). These results indicated developmental differences in the preschool period. Additionally, they showed that age 4 represents a shift marked by significant differences in the use of an argumentative strategy instead of the mere opposition to other’s point of view (Migdalek, Rosemberg, & Santibáñez Yáñez, 2014). Likewise, we have observed differences in the argumentative production of 4-year-old children according to the environment in which the play situation was taking place: preschool or the home (Migdalek, Rosemberg, & Arrúe, 2015).

## The Current Study

Considering the investigations which show that the social context may, to a greater or lesser degree, propitiate the use of argumentative strategies (Faigenbaum, 2012; Silvestri, 2001), in the present paper we aim to further delve into the Socio-Economic Status differences in children’s argumentative production. Moreover we will analyze children’s use of connectors in order to mark causal and adversative relationships within these arguments. To this end, we analyze a corpus of disputes during play situations in which 4-year-old socio-economically diverse Argentinean children participated at home.

Argentina is currently characterized by a fragmented social structure, reflected in great variations along dimensions of housing, occupation, and education. This determine markedly different living and developmental conditions for children growing up in the same city. In the city and in the metropolitan area of Buenos Aires there exist 1,102 informal settlements, *villas de emergencia*, which are marginalized urban shanty towns characterized by precarious housing, and insufficient or nonexistent infra-structure and services. Such neighborhoods are accessed by narrow dirt—or cement—floored corridors. Official data indicate that currently in these marginalized urban neighborhoods 400,900 families reside, approximately 2,004,500 people (Dirección General de Estadística y Censos, 2016). Most of them are descendants of indigenous populations who formerly resided in the North of Argentina or other countries of the region (such as Paraguay, Bolivia and Peru) and migrated to Buenos Aires in large numbers, in the late 20th century. The urban segregation of the villas de emergencia with respect to the residential neighborhoods, where middle and high income families, mostly from European origin, live, is accompanied by very marked differences in the level of education accessed by the population. This results in a major socio-economic inequality which is woven into socio-cultural but not linguistic differences, given that Spanish is the native language of the population.

With the objective of capturing the extremes of this variation we collected data of spontaneous argumentative discourse in the households of children living in villas de emergencias and in residential areas of the city of Buenos Aires.

## Method

### Design

This study follows a naturalistic approach (Guba & Lincoln, 1982; Teale, 1986): we analyzed situations of spontaneous interaction in mid and low SES households of 39 (23 females) 4-year-old Spanish-speaking children living in Argentina, specifically Buenos Aires City and surroundings.

### Participants

The 39 children were between the ages of 4:1 and 4:11. 19 of them belonged to families in which the parents had incomplete or complete primary education and live in villas de emergencia. The remaining 20 children belonged to families in which at least one of the parents had completed higher level education and lived in urban residential areas. Additionally, the parents differed in their occupation. According to report from the daycares’ personnel, all mid SES parents had stable employment as professionals. Low SES parents were either unemployed, had occupations requiring low levels of qualification (in domestic service or construction), and/or had unstable jobs as part of the informal labor market. In all cases they received government subsidies. In Argentina, it is not considered appropriate to ask families about their income, and thus this information was not available.

### Corpus

The data analyzed in this paper is part of a larger corpus recorded in an investigation about children’s linguistic and discursive development (Rosemberg, Arrúe, & Alam, 2005-2012).12 hours of natural interaction in everyday activities were recorded in each household (468 hours, 240 hs. in mid SES families and 228 in urban marginalized populations). The 12 hours were recorded in 3 or 4 observation days, which took place on different moments of the week and on different schedules. Adult participants and older children were asked to carry out their regular daily activities. The observer responded to the child’s and their family member’s comments, but did not encourage conversations or specific activities. The audio recordings were fully transcribed, the information was complemented with written annotations of non-verbal characteristics of the interactions (gestures, actions and objects employed, among others) and were included for later analysis. The whole process of data collection and transcription took an extended period of time, 7 years.

### Unit of Analysis

The units of analysis selected for this paper were the disputes between children and other participants in play situations. We chose to study argumentation in play situations specifically because play constitutes a governing and constitutive activity during the years of children’s lives. The disputes are defined as the meeting point of two confronting points of view, that are deployed in the interaction. The corpus of this study is made up of a total of 615 disputes: 453 of mid SES children and 162 of low SES.

### Procedure of Data Analysis

In order to analyze children’s argumentative strategies in the disputes, we follow a system of categories developed by Author and collaborators (Migdalek & Arrúe, 2013; Migdalek, Santibáñez Yáñez, & Rosemberg, 2014). This system was constructed through a mixed methodology that combines the use of a qualitative procedure (Grounded Theory: Glaser & Strauss, 1967; Strauss & Corbin, 1991) together with the heuristic use of categories developed in previous research. The system of analysis was aimed to conceptualize children’s argumentation skills from a perspective of cognitive and pragmatic development. Although we defined our categories based on previous research on adult argumentation (Perelman & Olbrechts Tyteca, 1989; Toulmin, 1958) as well as with the results of previous studies with children (Cobb-Moore et al., 2008, 2009; Dunn & Munn, 1987; Eisenberg, 1987), we adapted them in order to create a system capable of working with the specificity of our data (Migdalek, Santibáñez Yáñez, & Rosemberg, 2014).

To begin with, the system establishes a distinction between disputes in which the children only express their point of view and those in which they deploy some sort of argumentative strategy as support. Next, it gives account of the type of argumentative strategy, simple or complex, based on whether or not it delivers new information as an argument. The strategy of Reiteration was the only simple strategy identified. Likewise, for the quantitative study, we considered whether it was the main strategy for supporting the point of view (Reiteration 1) or whether it was a secondary strategy (Reiteration 2), that is, the reiteration of another argumentative strategy.

Complex strategies are divided into two large groups. One group is composed of strategies which select previous knowledge, structured in different formats, as a basis for the organization of the argumentative strategy. In this subgroup we include the Narration of previous experiences (referencing past events as evidence to support the point of view); Anticipation (the projection of courses of action whether desirable or not, connected to the point of view); Generalization (an affirmation which conceptualizes the phenomenon of the object of dispute with a certain degree of abstraction); and Description (the argument is organized from the selection and presentation of characteristics or properties of the object, an event or an internal state which allow speakers to assert their point of view as a conclusion).

The second group emphasizes the specific characteristics of the interlocutor as a basis for the strategy. Among these are the Appeal to Authority (the point of view is supported by the authority of the other interlocutor who has affirmed it previously); Politeness (the use of various linguistic resources which mitigate the formulation of the point of view so that it may be accepted); and the Alternative Proposal (a negotiation using some form of offer as a means to reach an agreement regarding the confronting positions in the dispute). Table 1 presents examples of each category.

**Table 1:** Examples of Argumentative Strategies

ES	EN
Reiteration	
Bruno: Ay, no, ¿por qué no puedo subir?	Bruno: Oh, no, why can‘t I climb up?
Franco: Vos subías y yo te tiraba, ¿dale?	Franco: You´ll climb up and I‘ll push you, ok?
Bruno: Sí, porque-	Bruno: Yes, because-
Franco: ¡No!	Franco: No!
Bruno: ¡Sí!	Bruno: Yes!
Franco: ¡No!	Franco: No!
Bruno: ¡Sí!	Bruno: Yes!
Franco: ¡No!	Franco: No.
Bruno: Sí.	Bruno: Yes.
Franco: No.	Franco: No.
Narration	
Carlitos: Está llorando tu pelota, Agu ¿Vamos a jugar vos con ella y yo con ella, dale, Agu? ¿Vos con ella, dale?	Carlitos: Your ball‘s crying Agu. Can you play with her and me with her, ok, Agu? You with her, ok?
Agustín: ¡No!	Agustín: No!
Carlitos: Y yo con esta.	Carlitos: And me with this one.
Agustín: ¡No!	Agustín: No!
Carlitos: ¿Por qué? ¿Con cuál querés vo?	Carlitos: Why? Which one do you want to play with?
Agustín: No porque vo me dejaste [pegaste] un pelotazo.	Agustín: No because you hit me hard with the ball.
Anticipation	
Carlitos: ¡Dale, Agus, juguemos ahora! Agustín: Con esta no, con esta no [se refiere a la pelota grande de cuero] si no me va a doler la cabeza, la panza.	Carlitos: C´mon, Agus, let´s play now! Agustín: Not with this one, not with this one con esta no [he refers to a big leather ball]. If not, it´s going to hurt my head, my tummy.
Generalization	
Carlitos: ¡Ahora la paso!	Carlitos: I´ll pass the ball in a second!
Agustín: ¡No, quiero pasarla, dale!	Agustín: No, I want to pass it, c´mon!
Carlitos: ¡No, esperá, yo la paso!	Carlitos: No, wait, I´m going to pass it!
Agustín: ¡No! Los jugadores [se refiere a los arqueros] no tienen que patear así parado, tiene que correr.	Agustín: No! Players [goalkeepers] can´t kick the ball standing, they must run.
Description	
Julieta: Mirá si se nos cae todo [se ríe].	Julieta: What if everything falls down? [Laugh].
Canela: Basta, no te rías porque me estoy desconcentrando.	Canela: Stop it, don´t laugh because I´m getting distracted.
Appeal to Authority	
Rafael: ¿Por qué no anda el control?	Rafael: Why isn´t the joystick working?
Lautaro: ¡Sí anda, Rafael!	Lauraro: It does work, Rafael.
Rafael: ¡El dos no anda!	Rafael: the number two doesn´t work.
LautaroI: ¡Lo dijo papá ayer!	Lautaro: Dad said it yesterday.
Politeness	
Cecilia: ¿Querés que- Clara, querés tus tortas?	Cecilia: Do you want- Clara, do you want your cakes?
Clara: [No responde]	Clara: [She doesn’t answer]
Cecilia: Tomá tu torta.	Cecilia: Here, take your cake.
Alternative Proposal	
Sol: Mirá, yo te presto el mío [un disfraz] y vos me prestás el tuyo.	Sol: Look, I´ll share mine [a costume] and you´ll share yours.
Guadalupe: Yo traje el mío para que yo me lo ponga.	Guadalupe: I brought mine so that [only] I can wear it.
Sol: ¡Dale!	Sol: Please!
Guadalupe: No, porque mamá y yo lo trajimos para que…	Guadalupe: No, because mom and I brought it so that…
Sol: Bueno, pero después...	Sol: OK, but afterwards…

The first author code the cent percent of disputes for both SES group. Twenty five percent of disputes of each SES group coded by the second author for reliability checking. Good indexes were found among the coders in all categories: Reiteration 1 (96.75% agreement, κ = .931), Reiteration 2 (96.10% agreement, κ = .729), Narration (97,40% agreement, κ = .843), Anticipation (98.05% agreement, κ = .943), Generalization (97.40% agreement, κ = .701), Description (92.20% agreement, κ = .783), Alternative proposal (98.70% agreement, κ = .850), Politeness (97.40% agreement, κ = .832), Appeal to authority (99.35% agreement, κ = .886).

We also analyzed the presence of certain linguistic resources—causal, consecutive and adversative connectors—used by the children in their argumentative production.

SES differences were statistically analyzed regarding the use of argumentative strategies, and in the presence of connectors. Given the differences in the quantity of disputes in both social groups, we calculated indexes considering the number of disputes each child was involved in.

## Results

Bearing in mind the investigations which show that the social context may foster the use of argumentative strategies (Faigenbaum, 2012; Kuhn, 1992; Silvestri, 2001), the present study aims to delve into the implications socio-economic differences might have on children’s argumentative production. To this end, we analyze a corpus of disputes in at-home play situations in which 4-year-old Argentine children from marginal urban communities and from mid SES families.

In the first place, we consider the degree to which children resort to some kind of argumentative strategy instead of simply expressing their point of view. We carried out a 2 x 2 variance mixed analysis, with SES (mid vs. low) as the inter-subject variable, and the use of strategies vs. the mere expression of a point of view as intra-subject variables. The post-hoc analysis was completed with the Bonferroni contrast test. The descriptive measures are shown on Table 2.

**Table 2:** Expression of Mere Point of View vs. Use of Argumentative Strategies by SES

Dispute structure	Mid SES		Low SES	
	*M*	*SD*	*M*	*SD*
Mere Expression of a Point of View	0.39	0.26	0.39	0.31
Use of an Argumentative Strategies	1.49	0.49	1.07	0.47

The variance analysis detects significant intra-group differences regarding the Use of an Argumentative Strategy to support a point of view and the Mere Expression of a Point of View *F*(1, 34) = 79.79, *p <* .001, in favor of using a particular strategy. The analysis also detects an effect of SES *F*(1, 34) = 5.81, *p <* .05: mid SES children use significantly more argumentative strategies. Likewise we detect an interaction effect between SES and the Use of an Argumentative Strategies vs. the Mere Expression of the Point of View *F*(1, 34) = 4.66, *p <* .05. Upon completion of the post-hoc analysis, we observed that SES renders no differences in the Mere Expression of a Point of View, but we did detect that mid SES children use significantly more argumentative strategies (*p <* .05).

Next we analyze the relationship between the type of argumentative strategies—focused on the use of evidence or on the interlocutor’s characteristics—and SES. Firstly, we carried out a mixed analysis of 2 x 2 variance, with SES as the inter-subject variable, and the type of strategies variable. Also a post-hoc analysis was carried out using Bonferroni’s contrast test. The statistical descriptives are shown on Table 3, below.

**Table 3:** Types of Argumentative Strategies by SES

Type of Argumentative Strategies	Mid SES		Low SES	
	*M*	*SD*	*M*	*SD*
Strategies centered on the use of evidence	0.96	0.50	0.38	0.28
Strategies centered upon the interlocutor’s characteristic	0.16	0.19	0.12	0.12

Without considering SES variation, results show significantly more use of argumentative strategies which imply the reference of evidence compared to those centered upon the interlocutor’s characteristics, *F*(1, 34) = 53.81, *p <* .001. Additionally, the analysis does not only detect SES effects *F*(1, 34) = 15.97; *p <* .001, (given the greater quantity of strategies in the mid SES group), but also an interaction effect between the social group and the type of strategy *F*(1, 34) = 13.97, *p <* .01. The post-hoc analysis showed that the differences appeared mainly in the strategies focused on the use of evidence, being employed to a greater extent in the mid SES group (*p <* .01).

**Figure 1:**
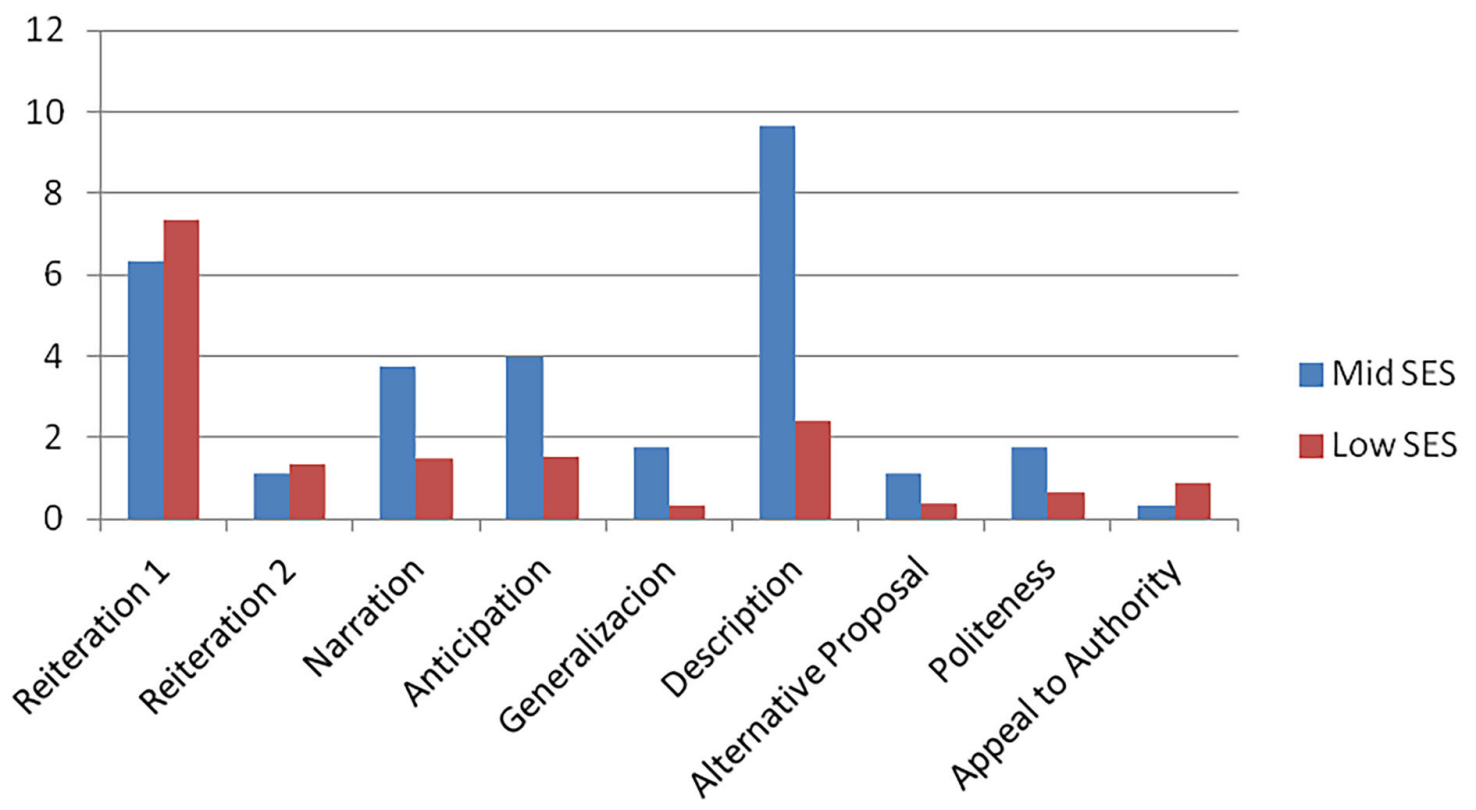
Distribution of argumentative strategies by SES.

Next we carried out an analysis of the argumentative strategies as a function of SES; distributional results are deployed in Figure 1. As observed in the Figure 1, low SES children resort to the strategy of Reiteration 1 (simple) most frequently, followed by the strategy of Description, then Narration and Anticipation in equal measure, Reiteration 2, Appeal to Authority, Politeness and, at the same level, Generalization and Alternative Proposal. For their part, mid SES children resort most frequently to Description, followed by Reiteration 1, then Anticipation, Narration, and next Generalization and Politeness in equal measure; then comes Reiteration 2 (complex) and finally an Appeal to Authority.

SES differences in the use of each of these strategies were assessed through the *t*-test. The descriptive statistics are shown on Table 4, below.

**Table 4:** Argumentative Strategies by SES

Argumentative Strategy	Mid SES		Low SES		*t*(34)	*p*
	*M*	*SD*	*M*	*SD*		
Reiteration 1	0.32	0.17	0.49	0.29	2.21	.03
Reiteration 2	0.06	0.06	0.08	0.12	0.83	.42
Narration	0.19	0.26	0.10	0.15	1.22	.23
Anticipation	0.20	0.29	0.10	0.13	1.24	.23
Generalization	0.09	0.09	0.02	0.05	2.89	.01
Description	0.48	0.22	0.15	0.20	4.60	< .001
Alternative Proposal	0.06	0.07	0.02	0.04	1.75	.09
Politeness	0.09	0.11	0.04	0.07	1.49	.15
Appeal to Authority	0.02	0.03	0.06	0.10	1.48	.16

Significant differences were found in the Reiteration 1 strategy: low SES children employed this strategy more frequently than mid SES *t*(34) = 2.21, *p <* .05. For their part, mid SES children resorted to the strategy of Generalization *t*(34) = 2.89, *p <* .01 and the strategy of Description significantly more often *t*(34) = 4.60, *p <* .001.

**Figure 2:**
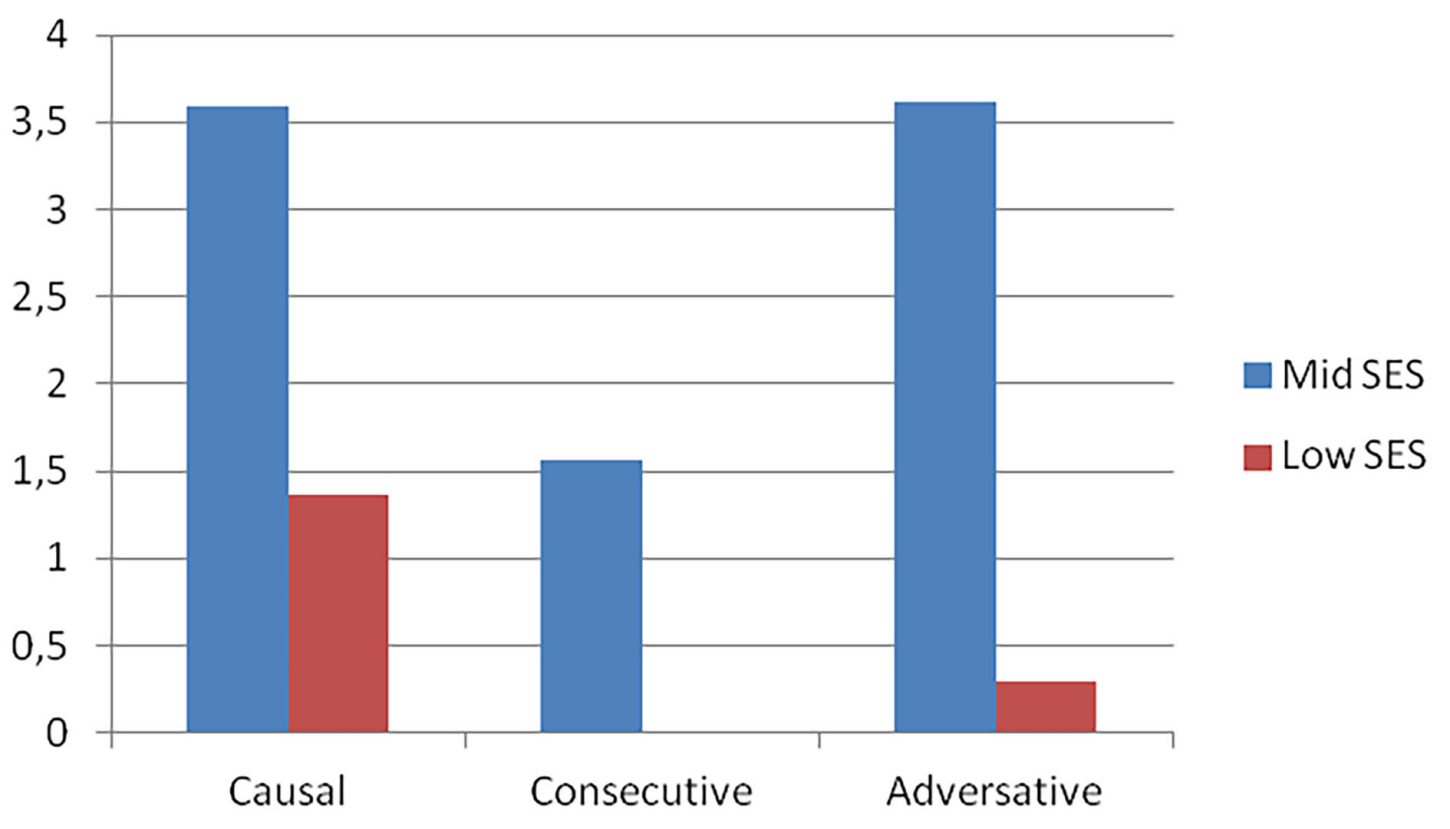
Distribution of connectors by SES.

Finally, we carried out an analysis of the connectors which were present in the disputes according to social group. As may be observed on Figure 2, the children from urban marginalized populations used causal connectors more frequently (0.9) than adversative connectors (0.2). In this group the use of consecutive connectors was not observed. For their part, the mid SES children also employed causal and adversative connectors to the same extent (0.18), followed by consecutive connectors (0.8).

SES differences regarding the use of each of type of connectors analyzed were assessed using the *t*-test. The descriptive statistics are below on Table 5.

**Table 5:** Use of Connectors by SES

Connector type	Mid SES		Low SES		*t*(34)	*p*
	*M*	*SD*	*M*	*SD*		
Causal connectors	0.18	0.21	0.09	0.12	1.61	.12
Consecutive connectors	0.08	0.14	0.00	0.00	2.44	.03
Adversative connectors	0.18	0.13	0.02	0.04	5.30	< .001

The analysis showed no significant SES differences in the use of causal connectors. Nonetheless, significant differences were found in the use of connectors to mark consecutive *t*(34) = 2.44, *p <* .05 and adversative links *t*(34) = 5.30, *p <* .001, as mid SES children used them significantly more frequently.

## Discussion

The results of the analysis of 4-year-old mid and low SES Argentinean children’s argumentative strategies produced during in at-home play situations presented in this paper provide additional evidence to previous research (Faigenbaum, 2012; Kuhn, 1992; Silvestri, 2001) regarding the importance of social context in early discursive acquisition. This study, differently from others (Pontecorvo & Arcidiacono, 2010), focused on only one type of activity —play—; therefore allowing for comparisons across social groups.

These results replicate the findings of our previous studies. Very early on, children from both social groups use argumentative strategies significantly more than simply opposing a point of view (Migdalek, Rosemberg, & Santibáñez Yáñez, 2014; Migdalek et al., 2015). In play situations, when required by the situational and/or discursive context, children are capable of producing diverse argumentative strategies showing experience and understanding of the social world, likely due to an early understanding of shared intentions, wishes and beliefs. These results also as do those of other studies (Dunn & Munn, 1987; Eisenberg, 1987; Peronard, 1991; Pontecorvo & Arcidiacono, 2010; Stein & Albro, 2001; Voss & Van Dyke, 2001; Zadunaisky Ehrlich & Blum-Kulka, 2010) highlight the early appearance of the argumentative use of language.

The analysis also showed an effect of social group: mid-SES children employed significantly more argumentative strategies in disputes than their low-SES peers, however the mere expression of point of view was observed to be equal across both groups. Additionally, mid SES children differed from their low SES peers, due to their more frequent use of the type of strategies that mainly relied on referencing evidence. As Nelson (1996, 2007) suggested at age 3 children make a more flexible use of their mental representations of usual events, as well as specific representations of past events, creating a cognitive context that allows them to understand and participate in the world. These knowledge representations are an important base from which they can draw on to create an argument. If their strategy is effective in a certain situation, this may well reinforce its subsequent usage, evincing a bi-directional relationship between cognition and social context.

In order to argue, low SES children employed the strategy of simple reiteration more than the mid SES group. This repetition resource has been studied in detail from classical to contemporary rhetoric (Perelman & Olbrechts Tyteca, 1989; Tindale, 1999). Other studies on child argumentation have also identified reiteration as a strategy found in children’s argumentation. It has been considered a simple argumentative strategy, given that it does not provide new information as an argument (Dunn & Munn, 1987; Eisenberg, 1987; Stein & Albro, 2001). It only requires the tacit recognition of a difference of opinion and insistence. It is difficult to assess whether low SES children resort mainly to reiteration because it is the more effective strategy in their context or due to the fact that their argumentative repertoire is limited. Alternatively, the combination of both hypothesis may better explain low SES children argumentative performance.

For their part, the mid SES group draws on the Generalization and Description strategies in a significantly greater number of opportunities than the low SES group. It is worth noting that in a previous study comparing low SES children’s argumentative performance in preschool versus at-home situations (Migdalek et al., 2015), we found a more frequent use of these strategies in the preschool context than at home.

These results provide new evidence to support the importance of context for deploying complex argumentative strategies (Faigenbaum, 2012; Kuhn, 1992; Silvestri, 2001). The use of the Generalization strategy may be attributed to the need to invoke social norms and rules that regulate interactions, especially at preschool (Cobb-Moore et al., 2008, 2009). Additionally, the taxonomic language that characterises school context from the start could explain the more frequent use of this strategy at preschool, as classroom interaction leads children to call upon superordinate concepts which subsume and provide explanations to specific concepts.

The analysis detected no significant SES differences in the use of causal connectors. As has been proved, these types of connectors, mainly the ‘because’ form, are used in disputes from an early age (Migdalek, Rosemberg, & Santibáñez Yáñez, 2014; Orsolini, 1993; Pellegrini, 1985). These results replicate Rosemberg, Silva and Stein (2010), who showed the early use of this connector in the production of narrative discourse by low SES children.

Conversely, the analysis did find significant differences in the use of consecutive and adversative connectors, being more frequent in the mid SES group. This could be due to mid SES children possessing a greater quantity and diversity of vocabulary, as has been shown in other studies (Rosemberg et al., 2014).

In short, the results of this study show important SES differences in the production of argumentative discourse. These differences may be attributed to variation in linguistic opportunities offered by social and discursive interactions. Furthermore, disputes during play situations have proved to challenge children to use linguistic resources and strategies in order to support their claims. Therefore, one pedagogical implication that arises from these results is that preschool teaching situations could be enriched by children’s spontaneous disputes, and become learning experiences with the potential of promoting argumentative discourse.
